# Establishment of the mild, moderate and severe dry eye models using three methods in rabbits

**DOI:** 10.1186/1471-2415-13-50

**Published:** 2013-10-05

**Authors:** Na Li, Xinguo Deng, Yang Gao, Shihua Zhang, Meifeng He, Dongqing Zhao

**Affiliations:** 1 State Key Laboratory of Ophthalmology, Zhongshan Ophthalmic Center, Sun Yat-sen University, 54 Xianlie Road South, Guangzhou 510060, China; 2 Huizhou First Hospital, 20 Sanxin Road South, Huizhou 516001, China

**Keywords:** Dry eye, Rabbit model, Conjunctival impression cytology, Corneal fluorescein staining, Schirmer I test

## Abstract

**Background:**

Dry eye (DE) is a common eye disease, and appropriate animal models are essential to explore the pathogenesis and therapy of DE. In this study, we aimed to establish rabbit models by three methods.

**Methods:**

In group A, the lacrimal gland, Harderian gland, and nictitating membrane of the left eyes were surgically removed. In group B, the bulbar conjunctiva of the left eyes was burned with 50% trichloroacetic acid. In group C, both methods above were used. The right eyes served as normal controls. The Schirmer I test (SIt), fluorescein staining, and impression cytology were evaluated at baseline and on days 28, 42, and 56.

**Results:**

Both the SIt and goblet cell density were significantly lower in operated eyes compared to the control eyes, while the corneal fluorescein staining scores in the operated eyes were significantly higher than in the control eyes on days 28, 42, and 56 (*p* < 0.05, *p* < 0.01 or *p* < 0.001). The SIt and goblet cell densities in groups B and C were significantly lower than group A on days 28, 42, and 56 (*p* < 0.05, *p* < 0.01 or *p* < 0.001). In addition, the corneal fluorescein staining scores in group C were significantly higher than either group A or group B on days 28, 42, and 56, while fluorescein staining scores were higher in group B than group A on days 42 and 56 days (*p* < 0.05, *p* < 0.01 or *p* < 0.001), with mean score 3.8 ± 1.30 (group A), 7.4 ± 1.14 (group B) and 10.8 ± 1.30 (group C) on day 56.

**Conclusions:**

Results suggest that three separate DE models, with mild, moderate, and severe manifestations of DE, could be stably established, in which conjunctival goblet cells took an important role.

## Background

Dry eye (DE), also referred to as keratoconjunctivitis sicca (KCS), is a multifactorial disease of the tears and ocular surface that cause discomfort, visual disturbance, and tear film instability, which can potentially damage the ocular surface. KCS is accompanied by increased osmolarity of the tear film and inflammation of the ocular surface [1]. The reported prevalence of DE ranges from 5% to 35%, and the condition is more prevalent in women and increases with age [2]. The clinical symptoms of DE vary from minor discomfort to dryness, a sensation of having a foreign body, asthenopia, photophobia, and pain, among others such as difficulty opening eyes and eyesight reduction. Together, these symptoms severely influence the quality of life for each patient [3,4]. Besides, several studies reported the impact of DE on vision related quality of life [5,6]. It is therefore prudent to explore the pathogenesis and therapy of DE. To achieve this, appropriate animal models of DE are essential.

Considering the complicated etiology, a huge variety of animal models are available to mimic the different pathophysiologic mechanisms of DE [7]. Some of those models include the hereditary mouse models resembling Sjögren’s syndrome [8,9], the mouse model induced by botulinum toxin B [10] or controlled environment [11], rat models induced by evoked dacryoadenitis [12] or anticholinergic drugs [13], rabbit models induced by closure of the meibomian gland orifices [14], controlled environment [15], evoked dacryoadenitis [16], preganglionic parasympathetic denervation [17], topical medication of a preservative [18], or removing of the lacrimal gland [19], canine models formed spontaneously [20] or induced by canine distemper virus [21], and monkey models by removing the lacrimal gland [22].

Because rabbits have large eyes amenable to slit-lamp microscopic examinations, and considering their gentle nature and relatively low cost to maintain, rabbit models are well-suited to study the development of DE. A common way to build a rabbit model of KCS is by disabling the lacrimal gland and surgically removing the Harderian gland and nictitating membrane simultaneously [23-25]. Another study [26] established a DE model in rabbits by burning the bulbar conjunctiva with 50% trichloroacetic acid then surgically removing the lacrimal gland, Harderian gland, and nictitating membrane. These reports, however, did not describe details regarding degrees of KCS. The purpose of our study was to establish the above two rabbit models of DE as well as a third rabbit model that involved burning the bulbar conjunctiva with 50% trichloroacetic acid only to compare the three different models.

## Methods

### Experimental animals and ethics statement

Fifteen female New Zealand white rabbits weighing 2.0–2.5 kg were obtained from the animal facility at the Sun Yat-sen University, China. Rabbits were reared under standard laboratory conditions (22 ± 2°C, 60% ± 10% relative humidity, and a 12-hour light–dark cycle). All rabbits had free access to food and water throughout the experiment. This study was performed in strict accordance with the ARVO Statement for the Use of Animals in Ophthalmic and Vision Research*.* The protocol was approved by the Committee on the Ethics of Animal Experiments of the Zhongshan Ophthalmic Center (Permit Number: 2008–014). All surgery was performed under general anesthesia by an intramuscular injection of 40 mg/kg ketamine hydrochloride, with every effort to minimize suffering.

### Grouping and induction of the DE models

**Figure 1:**
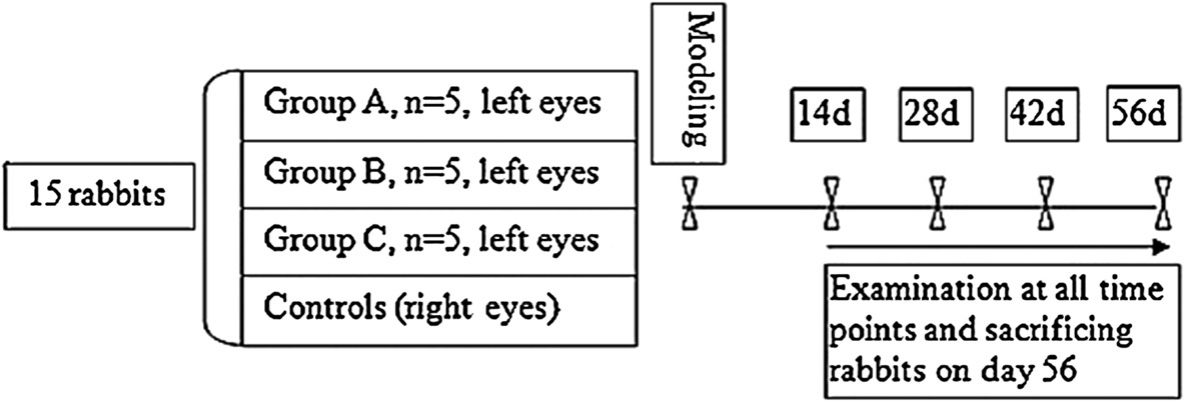
Experimental roadmap.

The 15 rabbits were randomly divided into three groups. Rabbits in Group A (n = 5) had the lacrimal glands, Harderian glands, and nictitating membranes of the left eyes surgically removed. In Group B (n = 5), the bulbar conjunctiva of the left eyes were burned with 50% trichloroacetic acid. In Group C, (n = 5), the bulbar conjunctiva of the left eyes was burned with 50% trichloroacetic acid and the lacrimal glands, Harderian glands, and nictitating membranes surgically removed (i.e., a combination of the two methods used in groups A and B). The right eyes served as the normal controls. See Figure 1.

All operations were performed under sterile conditions in the operating room for animals in Zhongshan Ophthalmic Center after washing the conjunctival sac with 0.9% sterile saline and sterilizing the surface around the eyes with lodophor. After instilling 0.5% proparacaine hydrochloride eye drops, a curve-shaped incision measuring 5 mm in length was created on the palpebral conjunctiva of the left eyes, 2 mm lateral to the palpebral margin. After blunt dissection of the intraorbital tissues and incising the fascia attached to the margin of the zygomatic arch, the lacrimal gland was gently dissected with curved smooth forceps. The removed glands had their complete capsules, and their volumes and lengths were similar as previously reported [27,28]. The nictitating membranes were snipped off at the base, and the Harderian glands, located between the medial rectus muscle and the anterior wall of orbit, were separated from the incision formed by excision of the nictitating membrane, were removed in their entirety, together with the complete capsule. By our way of removing the lacrimal glands and Harderian glands were simple, minimally invasive, and equally feasible. The lacrimal glands and Harderian glands were preserved for routine pathologic examination.

To burn the bulbar conjunctivas, a cotton swab soaked with freshly prepared 50% trichloroacetic acid was applied to the conjunctivas of the left eyes 2–3 mm lateral to the corneal limbus for 5 sec (when blanching of the conjunctiva was observed). The conjunctival sacs were immediately washed with 100 mL 0.9% sterile saline. Eye drops containing 0.3% tobramycin were applied 3 times daily for 3 days preoperatively. Eye drops containing 0.3% tobramycin and 0.1% dexamethasone 4 times daily as well as an eye ointment containing 0.3% tobramycin and 0.1% dexamethasone once a night for 7 days. All topical medications were applied to both eyes. On day 56 of the study, all rabbits were sacrificed with an overdose of anesthesia (chlorpromazine).

### Examinations

The Schirmer I test (SIt), corneal fluorescein staining, and conjunctival impression cytology were performed preoperative and again 14, 28, 42, and 56 days after establishing the model. All tests were administered by the same individual using standard techniques and under the same environmental conditions.

### Schirmer I test

Without instilling anesthetic drops, one Schirmer strips (Tianjin Jingming New Technological Development Co., Ltd, China) was inserted into the middle of the lower conjunctival sac and the rabbits’ eyes were closed. The length of the wetting (measured in mm) was recorded after 5 min. All tests were performed after the rabbits were attach to the fixation device and had grown quiet.

### Fluorescein staining

After instilling one drop of 1% fluorescein solution, the eyes of all animals were examined under the slit-lamp microscope (Topcon SL-D7, Tokyo, Japan.) with a cobalt blue filter at a magnification of 16. According to the grading standards of the corneal fluorescein staining described by Xiao et al. [29], the cornea was divided into four quadrants. The staining intensity in each quadrant was scored on a scale of 0–3 (for a maximum score of 12). Scoring was achieved using the following guidelines: no punctate staining on the cornea was assigned 0 points; punctate staining of 1–10 was assigned 1 point; punctate staining 11–30 was assigned 2 points; and, either punctate staining >30 or clumped staining was assigned 3 points. Scores ranging from 1 to 4 were defined as mild, scores from 5 to 8 were moderate, and scores from 9 to 12 were considered severe.

### Conjunctival impression cytology

After instilling 0.5% proparacaine hydrochloride eye drops and wiping away the excessive tear fluid, nitrocellulose (Sartorius AG.37070, Göttingen, Germany) filter paper with a pore size of 0.45 microns (cut into small discs with a diameter of 8 mm each) was gently placed on the surface of the superior temporal bulbar conjunctiva 2 mm lateral to the corneal limbus. Slight pressure was applied for 5–10 sec. The paper was then peeled off and immediately placed in freshly prepared 4% formaldehyde for at least 30 minutes. The papers were then stained with periodic acid-Schiff stain (PAS) according to the following protocol: (1) oxidation in periodic acid for 10 min, (2) rinsing in tap water for 5 min, (3) rinsing in distilled water once, (4) staining with fuchsin for 25 min, (5) reduction with sodium metabisulfite for 5 min, (6) rinsing in tap water for 5 min, (7) staining with hematoxylin for 5 min, (8) decoloring with 1% HC1-ethanol for 30 sec to 1 min, (9) rinsing in tap water for 5 min, (10) dehydration with 95% and 100% ethanol (1 min each), (11) vitrification by dimethylbenzene for 20 min, and (12) permanent mounting on slides. The morphology of cells was observed under a microscope (HB-10104A, Nikon Corp., Tokyo, Japan) with a 40× objective. All samples were assessed by the same individual. The density of the goblet cells was expressed as the average number of goblet cells/section in 10 different sections (at a magnification of 40×). Three specimens from each rabbit were evaluated.

### Light microscopy

After humane euthanasia, a strip of cornea and superior conjunctiva measuring approximately 5 mm in width vertically across the globe were removed from all rabbits and fixed in 10% formalin. After dehydration, the specimens were embedded in paraffin, cross-sectioned, and stained with hematoxylin and eosin (H&E). The morphology of the cornea and the conjunctiva was observed under a microscope with a 40× objective. The lacrimal glands and Harderian glands that were previously removed when establishing the DE models were also treated and examined as described above.

### Statistical analysis

The data were presented as mean ± standard deviation (SD). Statistical analysis was performed using SPSS version 16.0 (SPSS, Inc, Chicago, IL). A one-way analysis of variance followed by either the Bonferroni or Dunnett T3 test were used to compare outcomes at different time points within the same group and between the different groups at the same time point. A significance level was set at *p* < 0.05.

## Results

### Schirmer I test

Preoperatively, no significant difference in wetting length was identified between the left eyes and the normal controls (*p* > 0.05). On days 28, 42, and 56 of the study, the SIt was significantly lower than the preoperative measurements in both groups A and B. Similarly, the SIt was significantly less in group C at all times points (i.e., days 14, 28, 42, and 56 of the study; *p* < 0.01 or *p* < 0.001). However, there were no significant differences in SIt on days 28, 42, or 56 in each group (*p* > 0.05).

**Figure 2:**
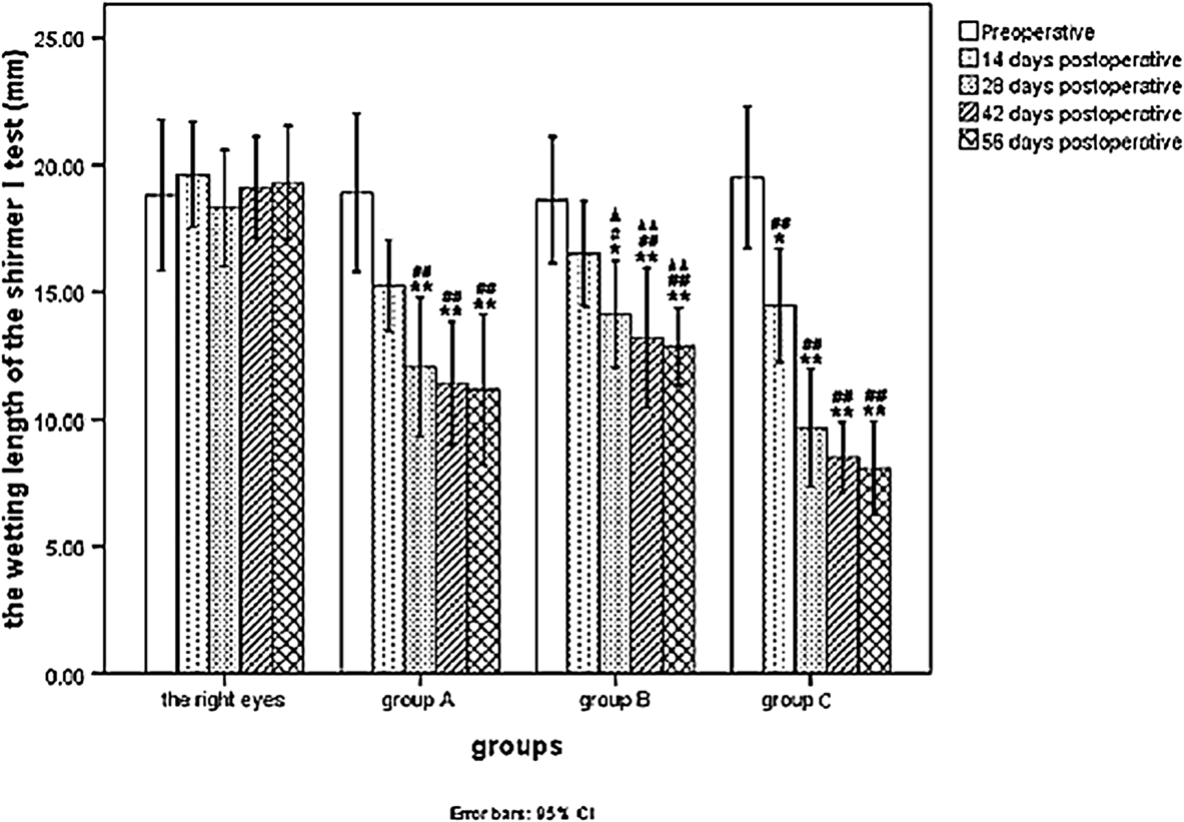
**Comparison of the wetting length of the shirmerItest at different time points between group A, group B and group C.** (*P < 0.01/**P < 0.001 vs. preoperative in the same group; #*P <* 0.05/##*P <* 0.001 vs. right eyes in all rabbits at the same time point; ▲*P <* 0.05/▲▲*P <* 0.001 vs. left eyes in rabbits of **group C** at the same time point).

As illustrated in Figure 2, the SIt in the left eyes of each group were significantly lower than in the control (right) eyes on days 28, 42, and 56. In group C, the SIt was also lower on day 14 (*p* < 0.05 or *p* < 0.001). The SIt in the left eyes of group C was significantly lower than group B on days 28, 42, and 56 (*p* < 0.05 or *p* < 0.001), but no significant difference between groups A and B were noted at any time point (*p* > 0.05).

### Corneal fluorescein staining

Slit-lamp examinations were performed at baseline then again every 14 days. Abnormal fluorescein staining of the cornea in the operated eyes in all the three groups was noted. There was no corneal punctate staining in any eyes prior to establishing the model, and no significant difference in scores was noted between the left and right eyes of each group preoperatively (*p* > 0.05). Compared to baseline, the corneal fluorescein staining scores in group A were significantly higher on days 28, 42, and 56. In groups B and C, fluorescein staining scores were higher on days 14, 28, 42, and 56 days (*p* < 0.05 or *p* < 0.01 or *p* < 0.001) compared to baseline. None difference in fluorescein staining scores were noted in the left eyes on days 42 and 56 in each group (*p* > 0.05).

**Figure 3:**
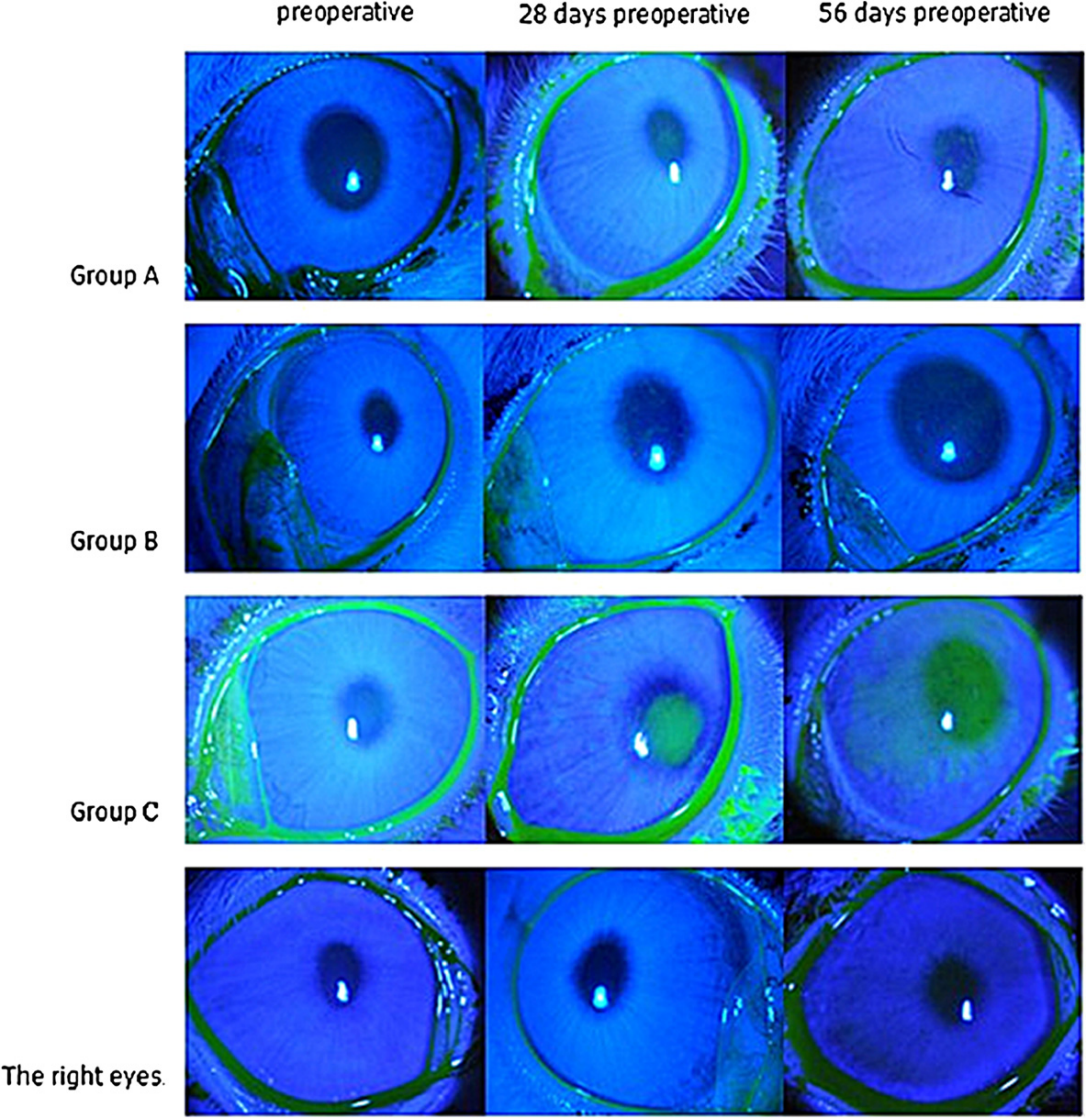
**Images of the corneal fluorescein staining (16×).** There was no punctate staining basically on the cornea of all eyes preoperative and right eyes at every time point postoperatively. The staining of the cornea in operated eyes increased gradually with time, and taken on different degrees in the three groups in the following order: **group A < group B < group C**.

**Figure 4:**
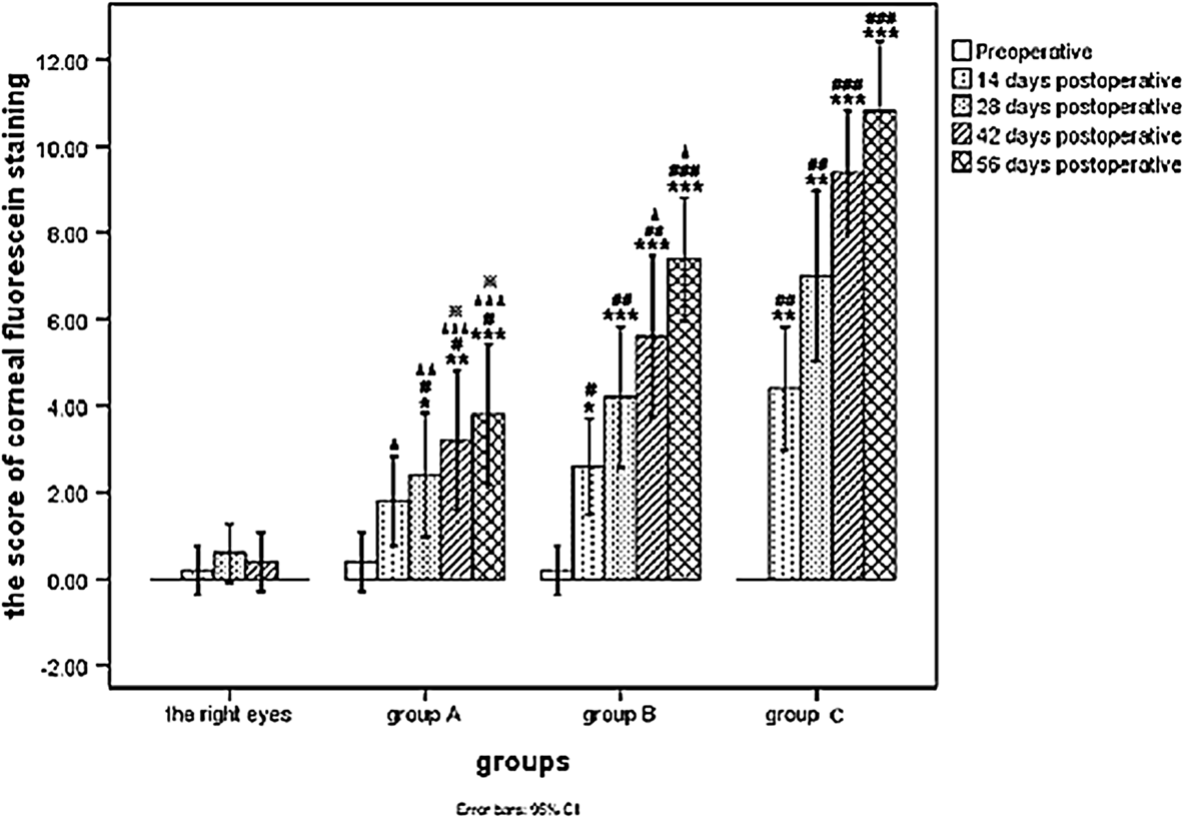
**Comparison of the score of corneal fluorescein staining at different time points between group A, group B and group C.** (*P < 0.05/**P < 0.01/***P < 0.001 vs. preoperative in the same group; #*P <* 0.05/##*P <* 0.01/###*P <* 0.001 vs. right eyes of all rabbits at the same time point;▲*P <* 0.05/▲▲*P <* 0.01/▲▲▲*P <* 0.001 vs. the operated eyes of rabbits in **group C** at the same time point; x※*P <* 0.01 vs. the operated eyes of rabbits in **group B** at the same time point.

Compared with the contralateral control (right) eyes, fluorescein staining scores were higher in the operated eyes on days 28, 42, and 56 in group A. In groups B and C, the scores were also significantly higher on day 14 (*p* < 0.05 or *p* < 0.01 or *p* < 0.001) compared with the control (right) eyes. The scores in the operated eyes of rabbits in group A were significantly lower than group C scores on days 14, 28, 42, and 56 days, and scores in group B were lower than group C on days 42 and 56 days (*p* < 0.05 or *p* < 0.01 or *p* < 0.001). Fluorescein staining scores of the operated eyes in group A was also significantly lower than group B scores on days 42 and 56 (*p* < 0.01). Thus, the mean corneal fluorescein staining scores of the rabbits in group A (3.8 ± 1.30), group B (7.4 ± 1.14), and group C (10.8 ± 1.30) were equivalent to a mild, moderate, and severe change at postoperative 56 days (Figures 3 and 4).

### Conjunctival impression cytology

**Figure 5:**
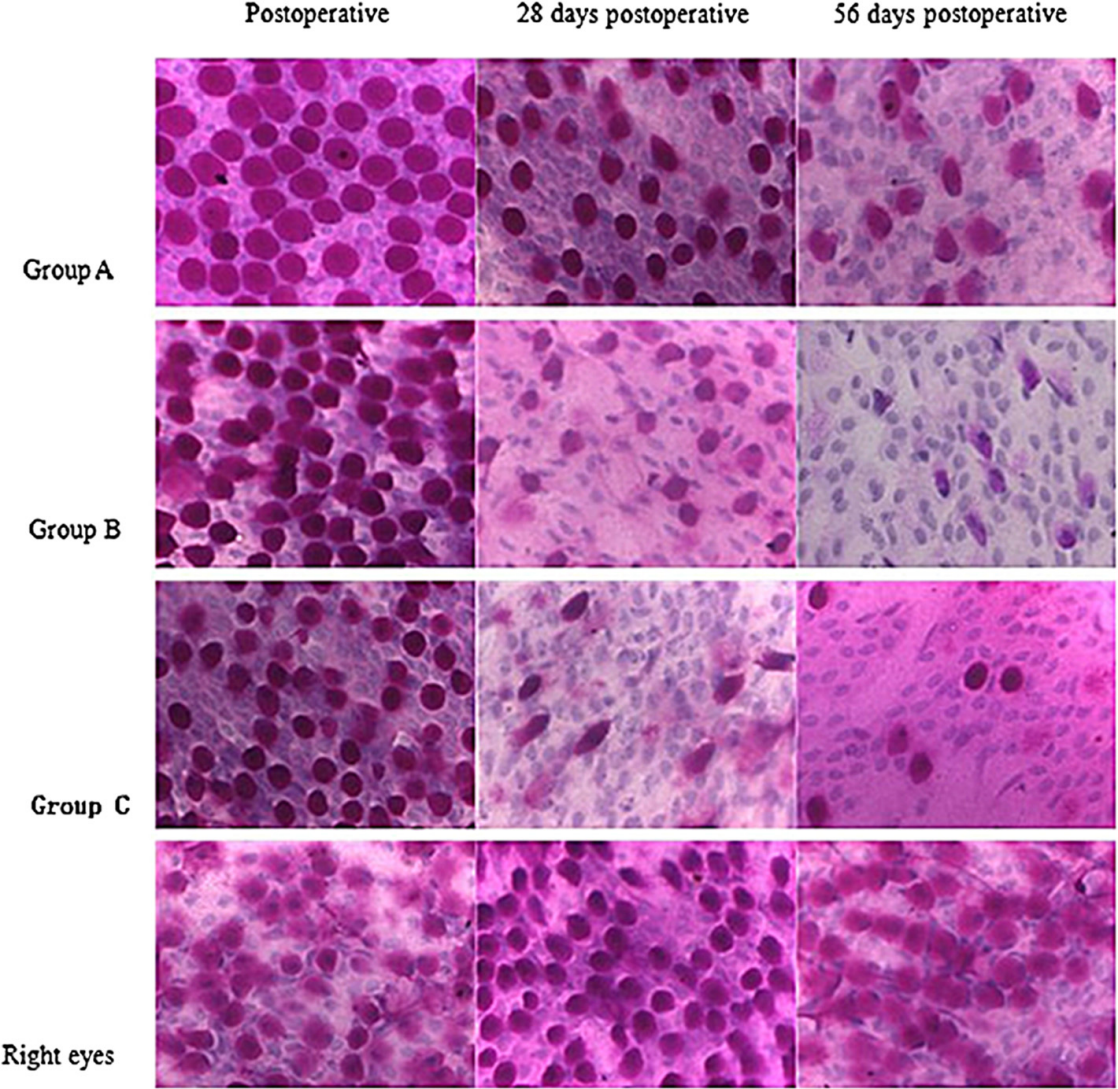
**Images of conjunctival impression cytology (CIC) (PAS, 400×).** Photographs of CIC in right eyes and left eyes preoperative showed a normal cytological image after PAS staining. After operation, the amount of goblet cells decreased and metaplasia of conjunctival epithelial cells turned up. Moreover, this trend was more severe in **group B** and **group C** than that in **group A**.

**Figure 6:**
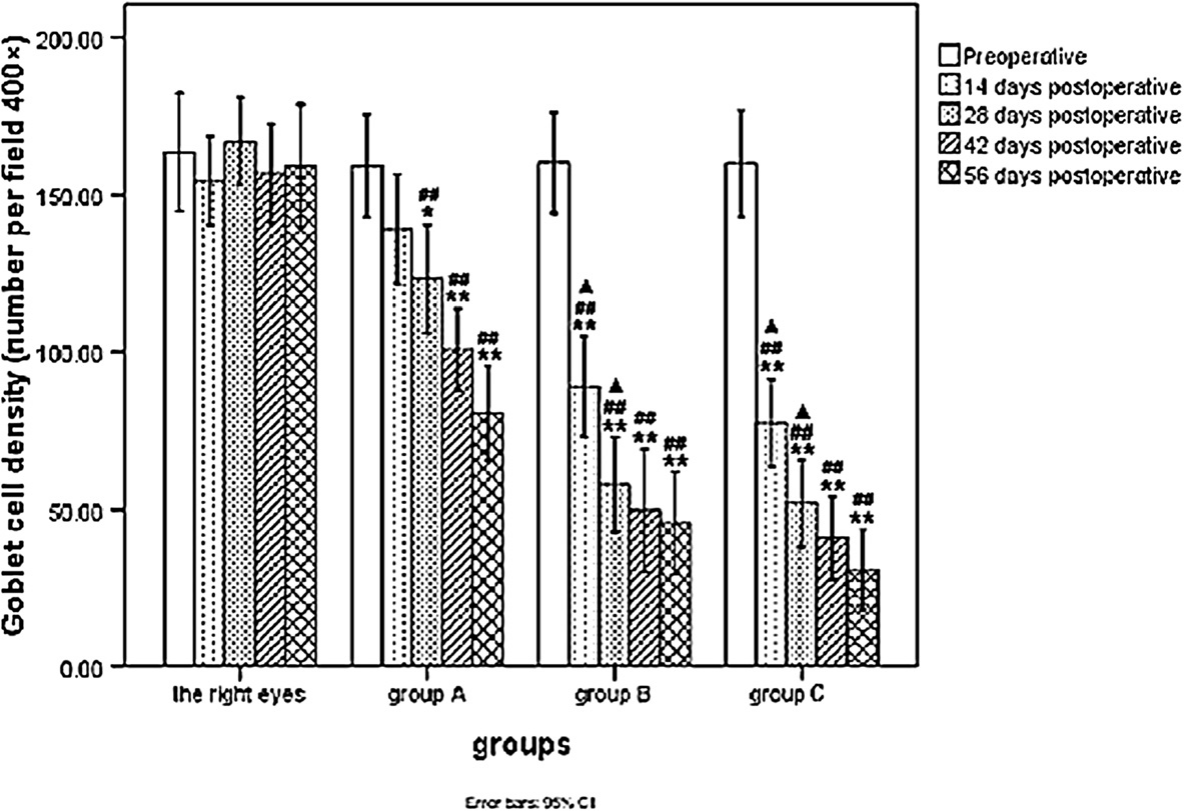
**Comparison of the score of conjunctival goblet cell density at different time points between group A, group B and group C.** (*P < 0.01/**P < 0.001 vs. preoperative in the same group; #*P <* 0.05/##*P <* 0.001 vs. right eyes of all rabbits at the same time point; ▲*P <* 0.001 vs. the operated eyes of rabbits in **group A** at the same time point).

Assessment of the conjunctival impression cytology focused on goblet cell density and metaplasia of corneal epithelial cells (Figures 5 and 6). No significant difference in goblet cell density was noted between the operated (left) and control (right) eyes of any group at baseline (*p* > 0.05). Goblet cell density in groups B and C was significantly lower on days 14, 28, 42, and 56 compared to baseline cell densities. Significant differences in goblet cell density were also lower in group A on days 28, 42, and 56 (*p* < 0.01 or *p* < 0.001) compared to baseline. There was no significance in goblet cell density on days 42 and 56 in each group (*p* > 0.05).

Compared with the contralateral control eyes, a significant decrease in goblet cell density was noted in the operated (left) eyes of each group on days 28, 42, and 56, and densities of the operated eyes were also significantly lower on day 14 in group B and group C compared to the controls (*p* < 0.05 or *p* < 0.001). Goblet cell density was significantly lower in the left eyes of rabbits in groups B and C than group A on days 14, 28, 42, and 56 days (*p <* 0.001).

### Light microscopy

**Figure 7:**
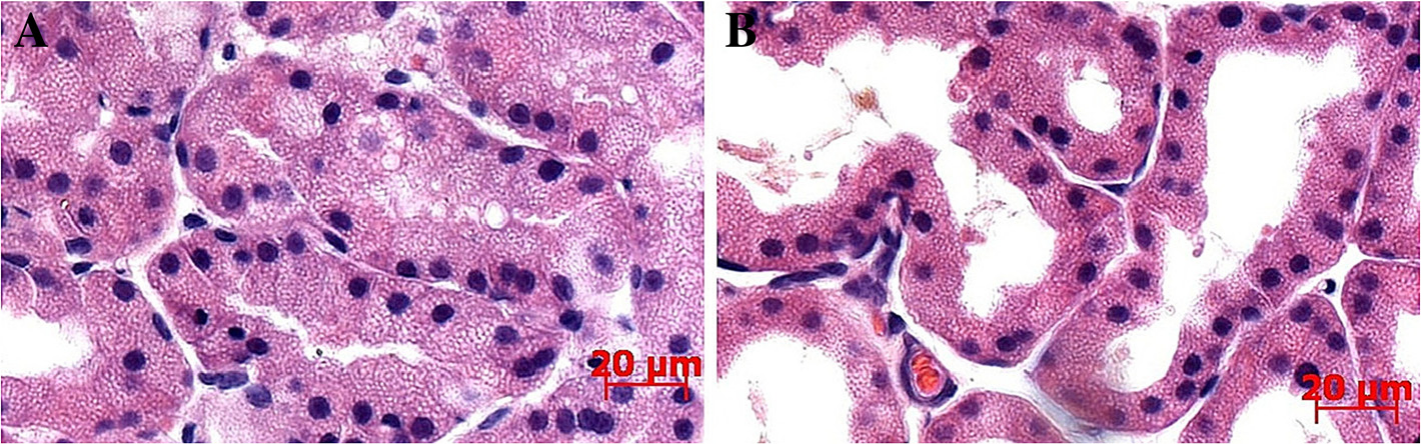
Characteristics of the lacrimal gland (A) and harderian gland (B) (HE staining, 400×).

The typical microscopic appearance of a normal lacrimal gland and Harderian gland has been provided in Figure 7 (H&E staining, 400×). The acinar epithelial cells of the lacrimal gland were columnar, with small round cell nuclei in the basal part and abundant vesicular mucus in the glandular lumens. Myoepithelial cells between the basement membrane and glandular epithelium were also evident, and the joints between the lacrimal gland acini were tight. The acinar epithelial cells of the Harderian glands were tall columnar, with round nuclei in the basal aspect. Myoepithelial cells between the basement membrane and glandular epithelium were also obvious, and the glandular lumens were large and irregular, within which vesicular mucus was noted. The joints between the Harderian gland acini were loose.

**Figure 8:**
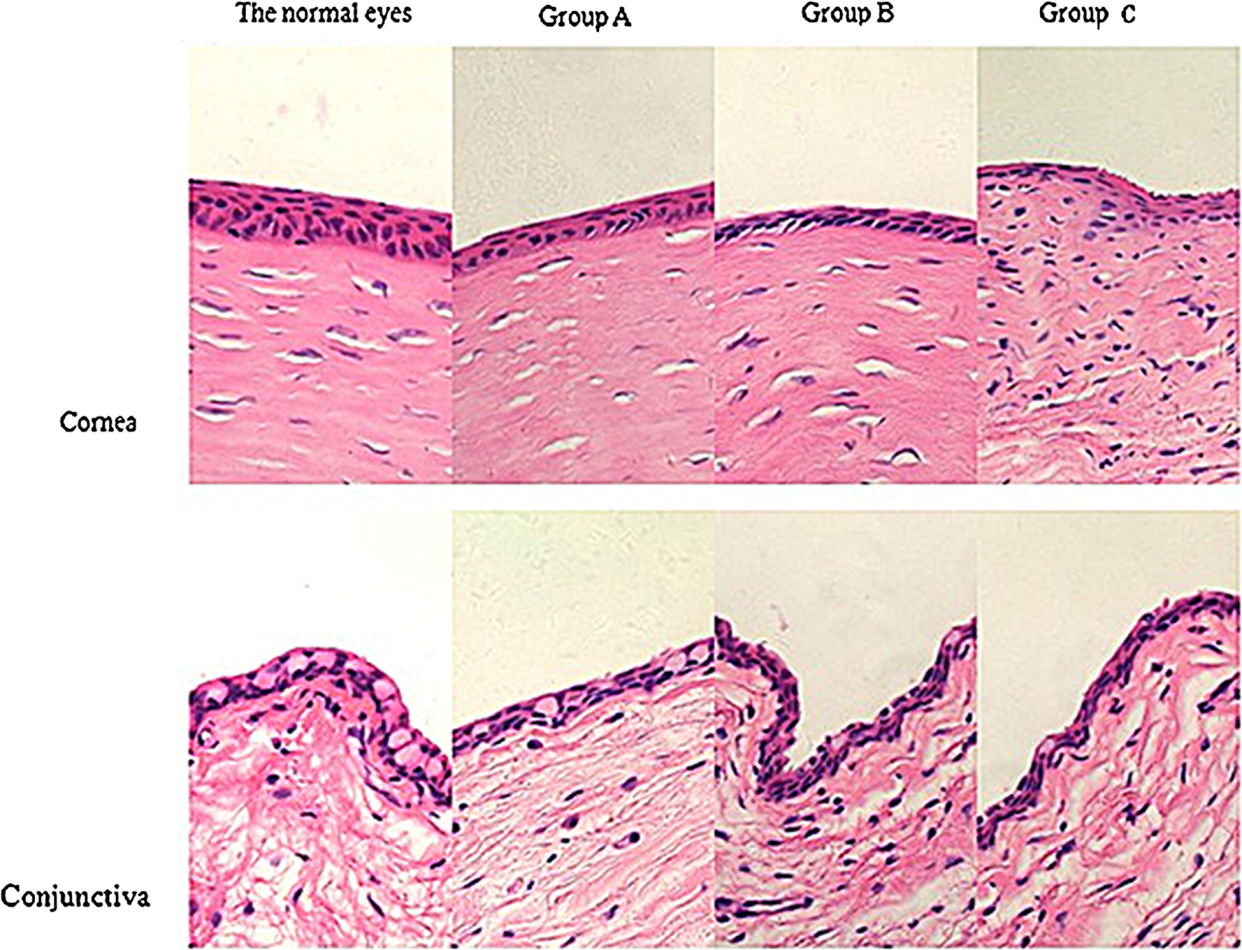
**Images of pathological changes in cornea and conjunctiva of rabbits in group A, group B and group C (HE, 400×).** In cornea of the operated eyes postoperative 56 days, thinner thickness and abnormal cellular morphology were observed, especially in **group C**. And infiltrations of inflammatory cells were observed in cornea of rabbits in **group C**. Besides, the number of conjunctival goblet cells was obviously decreased in the three groups, especially in **group B** and **group C**.

Pathologic changes in the cornea and conjunctiva of the operated (left) and control (right) eyes of rabbits in each group on day 56 have been described in Figure 8 (H&E staining, 400×). The cornea from the control eyes had a normal appearance in both thickness and cellular morphology. In the operated eyes, the corneas were thinner and abnormal cellular morphology was observed, which was particularly evident in group C. The surface of the cornea was rough, and an obvious increase in infiltration of inflammatory cells was noted in group C. The number of conjunctival goblet cells (the main change noted in the conjunctiva), was markedly decreased in all three groups, particularly groups B and C.

## Discussion

It is widely accepted that an essential role of the tear film is to provide a controlled environment for the ocular surface, comparable with blood circulation in the peripheral tissues. Any disruption or deficiency of the precorneal tear film can result in persistent discomfort with desiccation of the ocular surface. In rabbits, the tear film is approximately 6–7 μm thick between blinks. The tear film is structurally complex, with three distinct layers: a thin mucus layer on the surface of the corneal epithelium, a thick intermediate aqueous layer, and an extremely thin superficial lipid layer [30]. Under normal conditions, the lacrimal gland fluid contributes 58–79% of the total volume of tears in rabbits [31]. The secretory cells of the lacrimal gland produce a highly complex product of water (the chief component), ions, and proteins [32], which are the main components of the aqueous layer. The surface lipid layer is produced by both the meibomian glands and the Harderian glands [33], and the inner mucus layer is primarily produced by goblet cells in the conjunctiva, which played a key role in tear film “wetability” and stability [32,34]. Accessory lacrimal glands in the superior palpebral conjunctiva of rabbits are also present, which open onto the surface of the conjunctiva. The acinar systems of the accessory lacrimal glands have cellular features that resemble those of the main lacrimal glands [35]. In addition, the nictitating membrane can advance more than two-thirds of the distance across the eye, almost covering the cornea. The nictitating membrane therefore plays an important role in protecting the eyes of rabbits [27].

François et al. [23] established a rabbit model of DE by surgically removing the lacrimal gland, Harderian gland, and nictitating membrane. On the basis of this, Xie et al. [26] established a rabbit DE model by burning the superior and inferior fornix conjunctiva with 50% trichloroacetic acid and surgically removed the lacrimal gland, Harderian gland, and nictitating membrane. Those reports, however, did not described details regarding degrees of KCS. Instead, those reports only described the ultrastructural features of the cornea. Later, Gibard et al. [24,25] created DE models in rabbits by cauterizing the lacrimal gland excretory duct and surgically removing both the Harderian gland and nictitating membrane, which was similar to the method of François et al. Changes resembling DE in tear-film osmolarity, corneal epithelial glycogen, and conjunctival goblet cells were noted in the latter models; however, slit-lamp examination findings remained normal for the first 8 weeks after establishing the model.

In the current study, three DE models were established by destroying structures know to affect tear quality. Key findings were that the SIt and conjunctival goblet cell density were significantly lower in the operated (left) eyes of each group on days 28, 42, and 56 than in the control (right) eyes. Similarly, corneal fluorescein staining scores in each group were significantly higher in the operated eyes of each group on days 28, 42, and 56 than the control eyes. There were no differences in SIt, goblet cell density, or corneal fluorescein staining scores within each group on days 42 and 56 days. Thus, three separate rabbit models of KCS could not only be successfully established, but were also stable for at least 6–8 weeks. In addition, in group C, whose bulbar conjunctiva were burned with 50% trichloroacetic acid and the lacrimal glands, Harderian glands, and nictitating membranes were surgically removed, the DE model formed earlier and more severe than the other two models.

The SIt scores in group C were significantly lower than group B on days 28, 42, and 56, and goblet cell density in group C was significantly lower than group A on days 14, 28, 42, and 56. In addition, the corneal fluorescein staining scores in group C were significantly higher than both group A and B on days 28, 42, and 56, and the mean corneal fluorescein staining score in group C was 10.8 ± 1.30 on day 56, displaying severe corneal damage. Thus, the combined method used in group C not only greatly reduced the production of aqueous layer of tear film (primarily by the lacrimal gland), but also directly damaged the goblet cells on the conjunctiva, which is the main source of mucin in the tear film. In addition, the protective barrier of the ocular surface was damaged by removing the nictitating membrane, and lipid secretion was reduced, partly due to removal of the Harderian gland. The remaining tear fluid in rabbits in group C was likely only from fluid transported across the conjunctiva and/or fluid from the accessory lacrimal glands within the tarsal conjunctiva [35,36].

In group A (surgical removal of the lacrimal gland, Harderian gland, and nictitating membrane), significance changes in outcome measures were noted by day 28. This model primarily reduced tear production without directly damaging the conjunctival goblet cells. However, low lacrimal flow can cause tear hyperosmolarity, which indirectly (through activation of inflammatory pathways) may affect the density of goblet cells and/or goblet cell secretion [37]. The SIt results in group A were significantly lower than group B on days 28, 42, and 56 days, and goblet cell densities in group A were significantly higher than groups B and C on days 14, 28, 42, and 56, and corneal fluorescein staining scores in group A were lower postoperatively. The mean corneal fluorescein staining score in the rabbits of group A (3.8 ± 1.30) indicated only a mild lesions of the cornea. Therefore, this particular DE model could be successfully established mainly by reducing tear production, but in a mild degree, which may be related to either compensation of neurally regulated conjunctival goblet cell mucin secretion [36] or secretion of the accessory lacrimal gland on the conjunctiva.

In group B (burning the bulbar conjunctiva with 50% trichloroacetic acid only), lesions of the conjunctival goblet cells affected mucus production and tear film stability, which play a critical role in maintaining the health of the ocular surface and protecting it from environmental insults [36]. Compared with groups A and C, the SIt in group B was significantly higher on days 28, 42, and 56. This is because the lacrimal glands remained in good condition. In contrast, goblet cell densities in group B were significantly lower than group A on days 14, 28, 42, and 56. The corneal fluorescein staining scores in group B were significantly lower than both groups A and C on days 42 and 56. The mean corneal fluorescein staining score in group B (7.4 ± 1.14) indicated a moderate change by day 56. Those changes could be related to the destroyed goblet cells on the conjunctiva, which would greatly reduce mucus production and result in deficiency of the mucus layer and tear film unsteadiness. The inflammatory reaction evoked by poor condition of the ocular surface could contribute to the reduced tear production of the conjunctiva and cornea. Xiong et al. [18] successfully established a DE model in rabbits by topically applying a preservative, which primarily resulted in the destruction of the conjunctival goblet cells.

## Conclusions

This study reports the successful establishment of three different DE models in rabbits. A mild DE model established by surgically removing the lacrimal gland, Harderian gland, and nictitating membrane (group A), and a moderate DE model was established by burning the bulbar conjunctiva with 50% trichloroacetic acid (group B), in which conjunctival goblet cells took an important role. By combining above both techniques, a severe DE model was established (group C). All the three models were stable, and can be used to study DE for a variety of purposes.

## Competing interests

The authors declare that they have no competing interests.

## Authors’ contributions

NL carried out the examinations and operations of the study, and participated in analyzed the data and drafted the manuscript. XGD carried out the design and operations of the study and performed the statistical analysis. YG participated in the design and operations of the study. SHZ and MFH participated in its design and helped to draft the manuscript. DQZ participated in the examinations and operations of the study,and helped to draft the manuscript. All authors have read and approved the final manuscript.

## Pre-publication history

The pre-publication history for this paper can be accessed here:

http://www.biomedcentral.com/1471-2415/13/50/prepub
